# Post-antibiotic Ocular Surface Microbiome in Children: A Cluster-Randomized Trial

**DOI:** 10.1016/j.ophtha.2020.02.014

**Published:** 2020-08

**Authors:** Thuy Doan, Armin Hinterwirth, Lee Worden, Ahmed M. Arzika, Ramatou Maliki, Cindi Chen, Lina Zhong, Zhaoxia Zhou, Nisha R. Acharya, Travis C. Porco, Jeremy D. Keenan, Thomas M. Lietman

**Affiliations:** 1 Francis I Proctor Foundation, University of California San Francisco, San Francisco, California; 2 Department of Ophthalmology, University of California San Francisco, San Francisco, California; 3 The Carter Center, Niamey, Niger; 4 Department of Epidemiology and Biostatistics, University of California San Francisco, San Francisco, California; 5 Institute for Global Health Sciences, University of California San Francisco, San Francisco, California

Biannual mass oral administration of azithromycin to children decreases childhood mortality.^1^ These findings are associated with changes in the gut microbiome.^2^ Given that (1) the same biannual mass distribution of azithromycin is used for trachoma elimination, and (2) similar to the gut, the ocular surface is also lined with a mucous membrane, we sought to understand the effects of systemic antibiotics on the ocular surface of children in a cluster-randomized, double-masked, placebo-controlled trial.

Complete trial methods have been described.^1^^,^^2^ Briefly, this is a sister trial to Macrolides Oraux pour Réduire les Décès avec un Oeil sur la Résistance (MORDOR; clinicaltrials.gov NCT02048007), which is composed of 30 communities in Niger. Treatment randomization occurred at the community level, where children aged 1 to 59 months were randomized to receive 1 dose of oral azithromycin (target dose of 20 mg/kg) every 6 months or 1 dose of placebo every 6 months for 2 years (4 total treatments). A random sample of children in each community underwent conjunctival sample collection using a standardized protocol (Fig S1, available at www.aaojournal.org). Ethical approval was obtained from the University of California San Francisco Committee for Human Research and the Ethical Committee of the Niger Ministry of Health. The study was undertaken in accordance with the Declaration of Helsinki. Oral consent was obtained from guardians of children. Researchers processing and analyzing the samples were masked. Samples were processed as previously described.^2^ Ten samples from each community (300 samples at baseline and 300 samples at 24 months) were randomly chosen for pooling and metagenomic RNA sequencing.^2^

**Figure 1:**
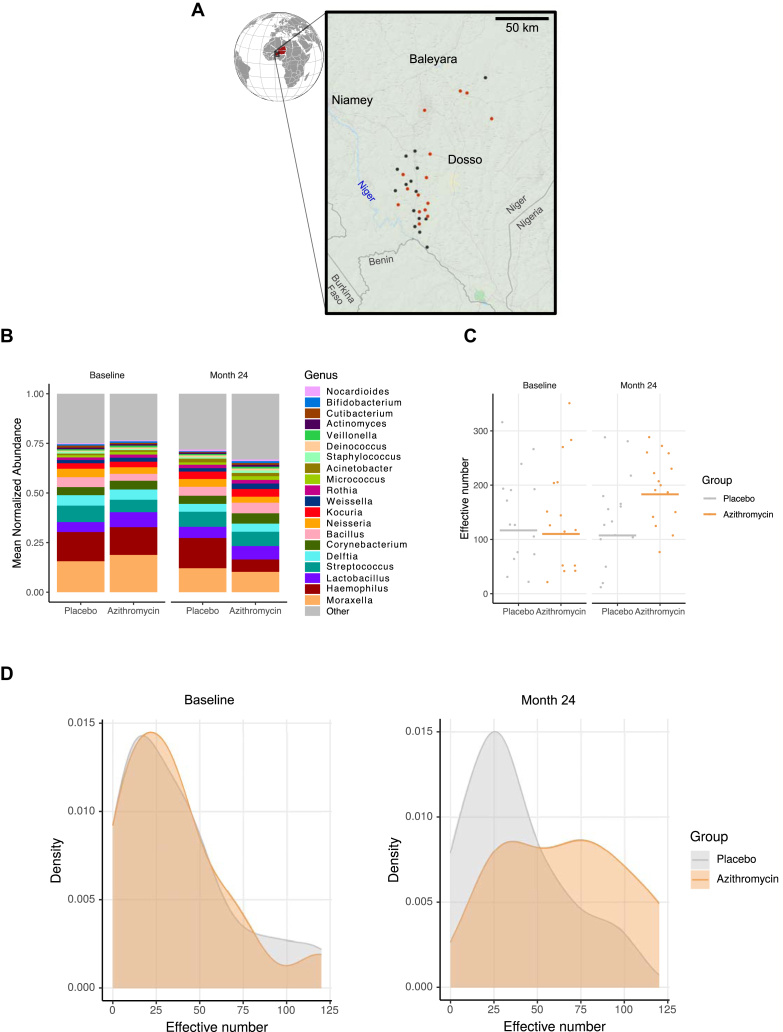
Ocular surface bacterial composition and diversity of children randomized to the placebo and azithromycin-treated groups. **A,** Locations of villages randomized in the Dosso region of Niger. **Black** and **red circles** represent villages randomized to placebo and azithromycin, respectively (terrain base maps www.thunderforest.com; data, www.osm.org/copyright, adapted under CC-BY-SA 2.0). **B,** Mean relative abundance of the 15 most abundant genera at baseline and 24 months for 600 samples from 30 villages between treatment groups. **C,** Scatter plots for Shannon’s diversity index at baseline (*P* = 0.84) and at 24 months (*P* = 0.04). **D,** Density plot for inverse Simpson’s diversity index at baseline (*P* = 0.99) and at 24 months (*P* = 0.02). All *P* values are permuted with 10 000 simulations.

The locations of the randomized communities are shown in Figure 1A. Age and gender between treatment groups are shown in Table S1 (available at www.aaojournal.org). *Haemophilus*, *Moraxella*, *Lactobacillus*, and *Streptococcus* were the predominant bacterial genera on the ocular surface (Fig 1B). The prespecified primary outcome was a change in the conjunctival bacterial community structures between arms. To determine a difference, we performed permutational multivariate analysis of variance (PERMANOVA) on the Euclidean distance (L^2^-norm) between the genus-level read numbers identified in each treatment arm. This outcome effectively assesses the distance between the centroids at the community level. At baseline (before treatment), there was no difference in the bacterial community structure (Euclidean PERMANOVA, *P* = 0.80). At 24 months (6 months after the fourth treatment), azithromycin treatment resulted in a significant change in the bacterial community structure (*P* = 0.03).

The prespecified secondary outcome was gamma diversity (estimated by alpha diversity of pooled samples) in units of effective number of species per sample. These community diversity values represent the richness of a community. At baseline, there was no difference in Shannon’s diversity (*P* = 0.84). We found that the diversity of ocular surface microbiome at the species level in the azithromycin arm (Shannon’s index, mean effective species number 183, 95% confidence interval [CI], 149–214) was higher than in the placebo arm (107, 95% CI, 55–152; *P* = 0.04, Fig 1C). Likewise, inverse Simpson’s diversity was also increased with azithromycin treatment (effective number 47, 95% CI, 34–63 vs. 12, 95% CI, 6–28, *P =* 0.02), whereas at baseline, there was no difference between arms (*P =* 0.99, Fig 1D).

Viruses are increasingly recognized as playing an important role in human mucosal health and immunity. In addition to its antimicrobial effects, azithromycin is suspected to have antiviral properties.^3^^,^^4^ The structure of the ocular surface virome was not altered after azithromycin treatment (Euclidean PERMANOVA, *P* = 0.93). Unlike the ocular surface bacterial community, the virome was sparse (mean effective species number 1.6, 95% CI, 1.2–2.6 for placebo and 2.0, 95% CI, 1.4–3.4 for azithromycin) and its diversity did not change with treatment (*P* = 0.78, inverse Simpson’s index, Fig S2A, available at www.aaojournal.org). Shannon’s index analysis yielded similar results (1.2, 95% CI, 1.0–1.8 vs. 1.3, 95% CI, 1.1–1.6, *P* = 0.79). The composition of the virome identified is shown in Figure S2B (available at www.aaojournal.org).

The richness in bacterial species detected in this study is similarly high compared with other deep sequencing studies of the human ocular surface.^5^^,^^6^ The multitude of organism sequences detected on the conjunctiva, however, may seem surprising given what is known about the ocular surface environment and physiology.^7^ The tear film that coats the ocular surface has innate antimicrobial properties and should be inhospitable for many pathogens. Furthermore, the number of cells obtained from a conjunctival swab is too few to account for the hundreds of organisms detected with high-throughput sequencing.^6^^,^^7^ Therefore, one possible explanation for the richness in microbial sequencing reads detected is that the majority of the microbial genera or species identified with high-throughput sequencing represents spillover of genetic material from adjacent sites, such as the skin, or mucosal sites, such as the oral cavity or respiratory tract, or even the environment, because the ocular surface is an exposed site. In children from countries where open defecation is common, the ocular surface microbiome may also include organisms from the gut, such as *Lactobacillus*. This spillover effect of genetic material from adjacent sites onto the ocular surface may be the reason for the observed increase in bacterial diversity with azithromycin distribution, although this hypothesis would need to be tested by concurrently swabbing and sequencing samples from adjacent sites. Generally, high microbial diversity is associated with health, whereas low diversity is associated with diseased states, and mass azithromycin distribution is shown to improve childhood mortality,^1^ presumably through improving the overall health of the treated communities.

The study’s interpretations may be limited to the study population in Niger. The samples are pooled, and the community diversity may not reflect what is found in an individual child. Likewise, antibiotic treatment at the community level reflects a herd effect and may not indicate the same effect when an individual child receives a single course of oral antibiotic. We studied children across a specific age range (1–59 months), and thus the results may be different in older children, adults, or even neonates given there is evidence to suggest that the microbiome matures over time. Finally, it is possible that acute changes occurred after azithromycin administration, but we were unable to capture those changes because the samples were collected 6 months after the fourth administration.

We showed that biannual mass distribution of azithromycin to children caused long-term alterations in the ocular surface bacterial community. Ocular functional relevance remains to be determined, although the same treatment resulted in a reduction in childhood mortality.^1^
