# Supplementary material for: Post-antibiotic Ocular Surface Microbiome in Children: A Cluster-Randomized Trial
Source: Ophthalmology. 2020 Aug;127(8):1127–30. doi: 10.1016/j.ophtha.2020.02.014 (PMC7384962; doi:10.1016/j.ophtha.2020.02.014)
Supplement: Figures S1 and S2 and Table S1 [file mmc1.pdf]

Supplementary Materials for

Post-antibiotic ocular surface microbiome in children: a cluster-randomized trial

Authors: Thuy Doan, MD, PhD[1,2*], Armin Hinterwirth, PhD[1], Lee Worden, PhD[1], Ahmed M. Arzika, MS[3], Ramatou Maliki, MPH[3], Cindi Chen, MS[1], Lina Zhong, BS[1], Zhaoxia Zhou, BS[1], Nisha R. Acharya, MD, MS[1,2], Travis C. Porco, PhD[1,2,4,5], Jeremy D. Keenan, MD, MPH[1,2], Thomas M. Lietman, MD[1,2,4,5]

Corresponding Author: Thuy.Doan@ucsf.edu

Figure S1

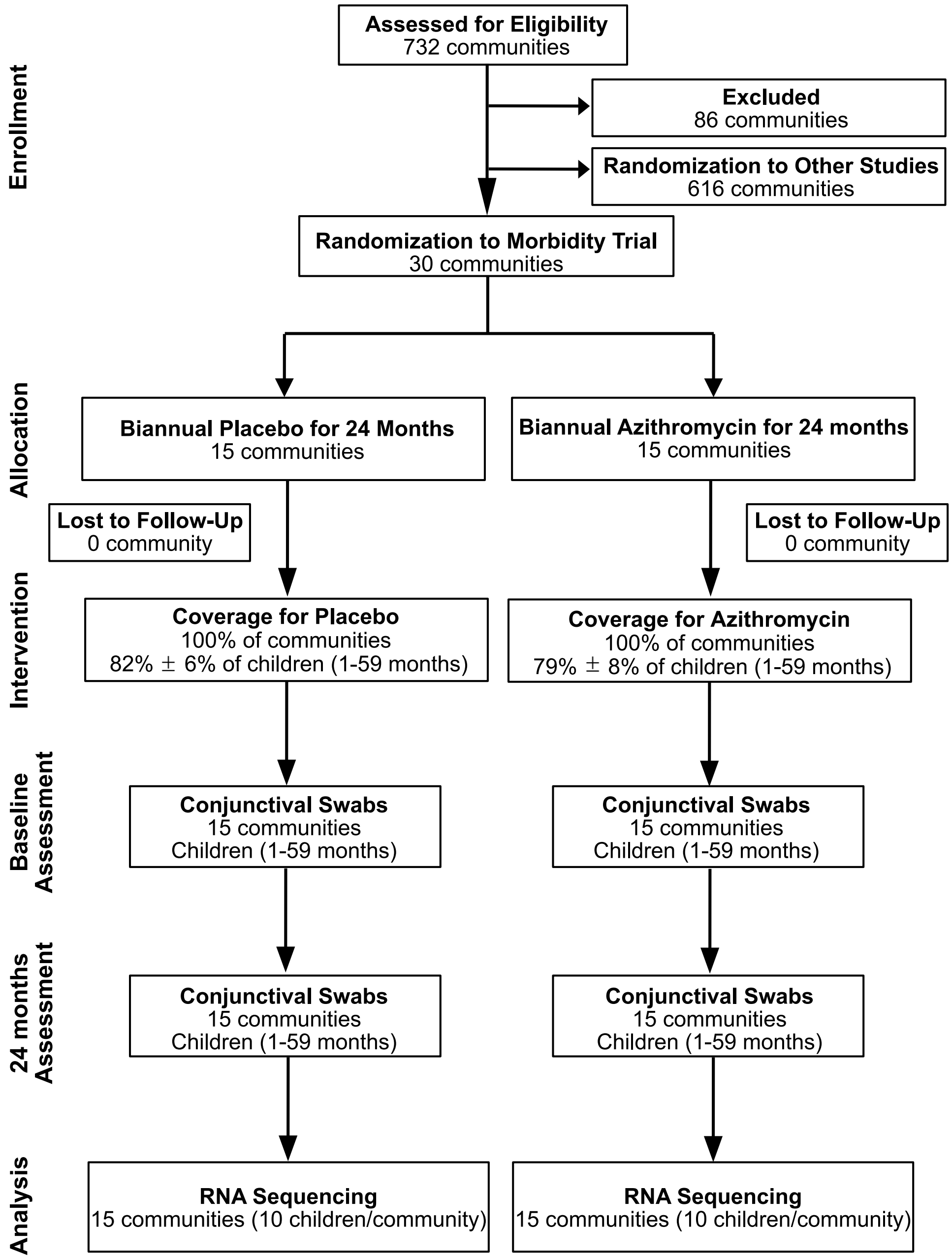

Figure S1. Study Design

Table S1: Demographics of Sampled Participants

	Conjunctival Swabs			
	Baseline		***24 months***	
	Placebo	Azithromycin	Placebo	Azithromycin
Number of children	150	150	150	150
Mean age, months (95% CI)	33 (31 to 35)	32 (30 to 34)	31 (29 to 33)	31 (29 to 33)
Age range, months	1 to 59	1 to 59	1 to 59	1 to 59
Female, % (95% CI)	39 (31 to 46)	54 (45 to 61)	51 (43 to 60)	47 (42 to 55)

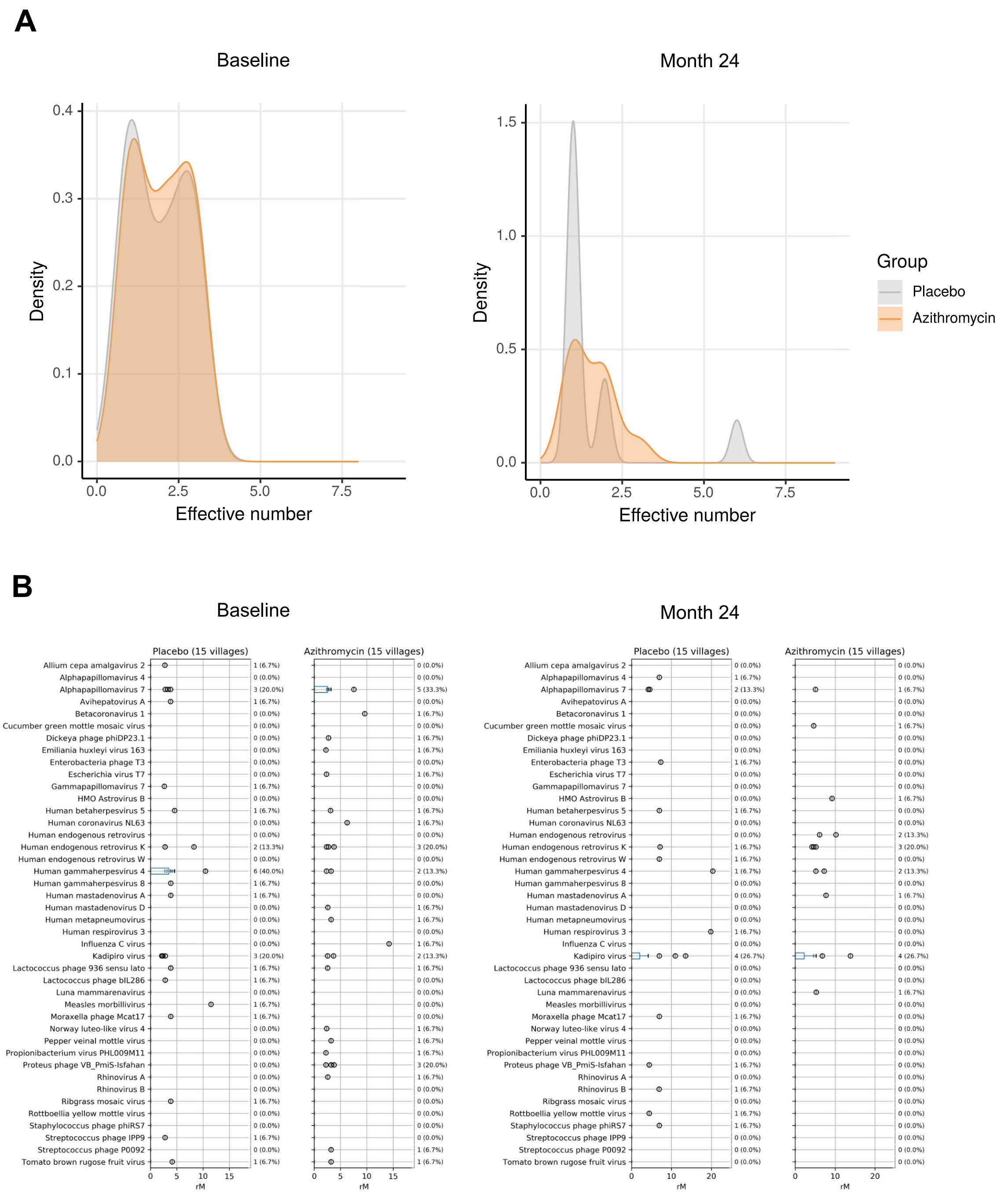

Figure S2. Ocular surface virome diversity and composition of children. (A) Density plots for inverse Simpson's diversity index at baseline (*P*=0.95) and at 24 months (*P*=0.78). (B) Viruses and phages detected across all villages at baseline and at 24 months. All *P* values are permuted with 10,000 simulations. Abbreviations: rM, reads per million reads.
